# The extent of postpartum cardiac reverse remodeling is reflected in urine proteome

**DOI:** 10.1038/s41598-024-65612-1

**Published:** 2024-06-27

**Authors:** Ana F. Ferreira, Fábio Trindade, Maria J. Azevedo, Juliana Morais, Thibaut Douché, Sílvia O. Diaz, Francisca A. Saraiva, Carla Sousa, Ana P. Machado, Mariette Matondo, Adelino Leite-Moreira, Carla Ramalho, Rui Vitorino, Inês Falcão-Pires, António S. Barros

**Affiliations:** 1 Cardiovascular R&D Centre – UnIC@RISE, Department of Surgery and Physiology, Faculty of Medicine of the University of Porto, Alameda Prof. Hernâni Monteiro, 4200-319 Porto, Portugal; 2 Faculdade de Medicina Dentária, Universidade do Porto, 4200-393 Porto, Portugal; 3 INEB - Instituto Nacional de Engenharia Biomédica, 4200-135 Porto, Portugal; 4 i3S - Instituto de Investigação e Inovação em Saúde, Universidade do Porto, 4200-135 Porto, Portugal; 5 Academic Center for Dentistry Amsterdam, University of Amsterdam and Vrije Universiteit Amsterdam, 1081 LA Amsterdam, The Netherlands; 6 Proteomic Platform, Mass Spectrometry for Biology Unit, CNRS UAR 2024, Institut Pasteur, Université Paris Cité, 75015 Paris, France; 7 Cardiology Department, Centro Hospitalar Universitário de São João, 4200-319 Porto, Portugal; 8 Center of Prenatal Diagnosis, Obstetrics Department, Centro Hospitalar Universitário de São João, 4200-319 Porto, Portugal; 9 Cardiothoracic Surgery Department, Centro Hospitalar Universitário de São João, 4200-319 Porto, Portugal; 10 Obstetrics, Gynaecology and Pediatrics Department, Faculty of Medicine of the University of Porto, 4200-319 Porto, Portugal

**Keywords:** Pregnancy, Cardiovascular reverse remodeling, Cardiovascular risk factor, Hemodynamic overload, Urine, Proteome, Biomarkers, Cardiology, Medical research, Molecular medicine, Risk factors

## Abstract

The association of postpartum cardiac reverse remodeling (RR) with urinary proteome, particularly in pregnant women with cardiovascular (CV) risk factors who show long-term increased risk of cardiovascular disease and mortality is unknown. We aim to profile the urinary proteome in pregnant women with/without CV risk factors to identify proteins associated with postpartum RR. Our study included a prospective cohort of 32 healthy and 27 obese and/or hypertensive and/or diabetic pregnant women who underwent transthoracic echocardiography, pulse-wave-velocity, and urine collection at the 3rd trimester and 6 months postpartum. Shotgun HPLC–MS/MS profiled proteins. Generalized linear mixed-effects models were used to identify associations between urinary proteins and left ventricle mass (LVM), a surrogate of RR. An increase in arterial stiffness was documented from 3rd trimester to 6 months after delivery, being significantly elevated in women with CV risk factors. In addition, the presence of at least one CV risk factor was associated with worse LVM RR. We identified 6 and 11 proteins associated with high and low LVM regression, respectively. These proteins were functionally linked with insulin-like growth factor (IGF) transport and uptake regulation by IGF binding-proteins, platelet activation, signaling and aggregation and the immune system’s activity. The concentration of IGF-1 in urine samples was associated with low LVM regression after delivery. Urinary proteome showed a predicting potential for identifying pregnant women with incomplete postpartum RR.

## Introduction

Pregnancy induces significant multisystemic changes to ensure a successful adaptation to fetal growth and development. Pregnancy’s cardiovascular response to blood volume expansion triggers an increase in preload accompanied by a reduction of peripheral vascular stiffness and a subsequent compensatory decline in afterload^1,2^. These circulatory adaptations induce cardiac remodeling, involving non-pathological left ventricle (LV) eccentric hypertrophy and slight diastolic dysfunction with left atrium (LA) enlargement^1,3^ but with preserved ejection fraction^1,3^. This physiological cardiovascular (CV) remodeling may be influenced by CV risk factors, such as arterial hypertension, gestational diabetes and obesity. Indeed, the CV remodeling in pregnant women with any of these CV risk factors is characterized by diastolic dysfunction combined with an increased left ventricular mass (LVM) and increased systemic vascular resistances^4,5^. Some authors suggest that women’s heart fully recovers without cardiovascular complications during gestation and that global and segmental myocardial performances return to their pre-gravid state/condition in a process known as reverse remodeling (RR)^2,3^. Although it has been reported that pregnant women with CV risk factors have an increased risk for potential cardiovascular disease and mortality in the long term, few studies have explored their impact on cardiac RR during postpartum^6,7^. We hypothesized that cardiac RR after delivery is associated with distinct proteomic traits in pregnant women with and without CV risk factors and that these are mirrored in urine. As a plasma filtrate, urine can be viewed as a vast collection of products from cardiovascular activity. Therefore, it should harbor many proteins potentially differentiating physiological from pathological cardiac RR. Besides, urine analysis brings additional advantages when compared to plasma/serum, including sample stability (the bulk of proteolytic activity is completed in the bladder), lower matrix complexity (protein dynamic range in urine is about half of plasma’s), and the non-invasive collection^8^. Urine proteomics allows for uncovering potential markers and for developing predictive tools, such as the recently reported Heart Failure Predictor, a classifier based on 96 urinary peptides^9^. Identifying pregnant women with a higher risk of a pathological cardiac RR is important to establish a tight monitoring program and eventually extend the follow-up until full cardiac RR is attained. Thus, this study aimed to profile the urinary proteome in pregnant women with or without CV risk factors and identify putative proteins associated with cardiac postpartum RR.

## Methods

### Study design and participants recruitment

We designed a prospective cohort study^10^, whose participants were recruited at the Department of Obstetrics of *Centro Hospitalar Universitário São João (CHUSJ)* and *Unidade Local de Saúde de Matosinhos—Hospital Pedro Hispano (ULSM-HPH)*. The Ethics Committees of CHUSJ and ULSM-HPH approved the study (ID 201/18 and ID 154/20/RS, respectively). All included participants provided written informed consent. The confidentiality and data anonymity complied with the Declaration of Helsinki.

Participants were recruited from March 2019 to March 2021 at their first medical appointment (in the 1st or 3rd trimester of pregnancy) or voluntarily through online forms available at https://perimyrobb.wordpress.com/.

The inclusion criteria were adult pregnant women (> 18 years old) with or without CV risk factors, namely chronic and/or gestational hypertension and/or gestational diabetes mellitus (DM) or type-2 DM, and/or obesity. Arterial hypertension was defined as systolic blood pressure (SBP) ≥ 140 mmHg and/or diastolic blood pressure (DBP) ≥ 90 mmHg measured in office or in-hospital before 20 weeks of gestation. Gestational hypertension was defined as arterial hypertension diagnosed after 20 weeks of gestation and resolving within 42 days postpartum. Gestational DM was diagnosed if 92 ≤ fasting-glucose ≥ 126 mg/dL at 1st trimester or ≥ 180 mg/dL or ≥ 153 mg/dL, respectively, 1 or 2 h after an oral glucose tolerance test (75 g oral glucose load) performed at 24–28 pregnancy weeks. Type-2 DM was diagnosed through the following criteria: glycated haemoglobin ≥ 6.5% or fasting plasma glucose ≥ 126 mg/dL or two-hour post-load venous plasma glucose ≥ 200 mg/dL after oral glucose tolerance test. Obesity was considered if the body mass index ≥ 30 kg/m^2^ before pregnancy.

Women with twin pregnancies, pre-existing cardiomyopathy, renal disease, chronic obstructive airway disease, active systemic infection, genetic syndromes or type-1 diabetes *mellitus* were excluded.

Two study groups were set according to the presence or absence of CV risk factors defined above, resulting in the definition of a healthy group and an HDO group (which included any combination of hypertensive, diabetic and obese women).

### Measurements

In the 3rd trimester (30–35 weeks), at the peak of cardiac remodeling (when cardiovascular adaptations are noticeable) and 6–7 months after delivery (during RR), participants underwent clinical characterization (maternal cardiovascular health, health-related habits, smoking habits, parity, medical history, demographics, obstetric and perinatal outcomes were obtained from questionnaires or electronic medical records), transthoracic echocardiography, pulse wave velocity (PWV) measurement and urine collection. The LVM was defined as the surrogate for assessing cardiac RR.

### Echocardiographic assessment

Conventional transthoracic echocardiography evaluation was performed with a 3 MHz phased-array probe (ACUSON SC2000 PRIME™) by a single operator and measurements obtained from standard views according to European Society of Cardiology recommendations for chamber quantification and diastolic function evaluation (Supplementary Table 1)^11,12^. At least three cardiac cycle images were acquired for data analysis. Two certified cardiologists independently analyzed, interpreted and harmonized the results. Myocardial deformation was assessed in LV through strain and strain rate analysis by Syngo Velocity Vector Imaging software, version 3.5 (Siemens Healthcare, Erlangen, Germany).

### Vascular stiffness evaluation

After 10 min of rest, brachial blood pressure was measured. Complior® device (Alam Medical, France) quantified arterial stiffness by carotid-femoral PWV, calculated from carotid-femoral distance/transit time. The mean value of two PWV measurements (whose difference was ≤ 0.5 m/s) were performed in each session for each participant.

### Urinary proteomic profiling

Fifty milliliters of first-morning urine samples were collected, centrifuged (2370 × g, 15 min, 4 °C), and stored at − 80 °C in each visit (3 T and 6 months after delivery). Protein was concentrated using 10 kDa-cutoff concentrators (Vivaspin 500, Sartorius Biotech, Göttingen, Germany). The protein retentate was solubilized in 0.3 M Tris pH 6.8 and 4% sodium dodecyl sulphate and quantified by a standard bicinchoninic acid assay (Pierce, ThermoFisher™, Rockford, IL, USA).

The urinary proteome was profiled by a label-free shotgun approach. Briefly, 100 µg of protein was incubated 1 h at RT in the dark for denaturation, reduction, and cysteine alkylation using a homogenization buffer composed of SDS 1%, TCEP 5 mM and chloroacetamide 20 mM in ammonium bicarbonate 50 mM, pH 8.

Protein digestion was carried out on an auto Single-Pot Solid-Phase-enhanced Sample Preparation (auto-SP3) on the Bravo (Agilent technologies) platform, with trypsin, for 12 h at 37 °C. Peptides were cleaned up using C18 cartridge (Agilent Technologies,), vacuum-dried and resuspended in acetonitrile (ACN) 2%, formic acid (FA) 0.1% buffer.

The peptides were separated and identified through a reverse-ohase nanochromatographic system (Proxeon EASY-nLC 1200-Thermo Fisher Scientific) coupled to a Q Exactive Plus mass spectrometer (Thermo Fisher Scientific). Peptides were eluted with a multi-step gradient over 118 min. Mass spectra were acquired using Xcalibur software using a data-dependent Top 10 method with a survey scan (300–1700 m/z) at a resolution of 70,000 and MS/MS scans at a resolution of 17,500.

Raw data were analyzed using MaxQuant software version 2.0.3.0 using the Andromeda search engine. The MS/MS spectra were searched against a UniProt *Homo sapiens* database (download in 13/12/2021). One unique peptide to the protein group was required for the protein identification. For quantification, we only considered protein groups with two unique peptides. A false discovery rate (FDR) cutoff of 1% was applied at the peptide and protein levels.

Proteins showing to be independently associated with cardiac RR were subjected to protein–protein interaction and functional enrichment analyses with STRING (version 11.5) webtool (accession date: 28^th^ March 2023). A score of 0.4 was set as the minimum threshold for consideration of the validated and putative interactions.

### Quantification of insulin-like growth factor-1 through ELISA assay kit

Insulin-like growth factor-1 (IGF-1, DY291, R&D Systems) was quantified in pairwise urine samples collected 3 T and 6 months after delivery. Three operators, blinded to clinical and echocardiographic information, assessed this ELISA assay kit on each urine sample using a commercially available kit following the manufacturer's instructions.

### Statistical analysis

Clinical parameters, physical assessment, echocardiographic and PWV values, and urinary proteins detected and quantified were gathered on a database and analyzed using *lmer*, *ggplot2* and *gtsummary* R packages, in the R statistical software environment version 4.2.1.

Continuous variables are expressed as median, 1st and 3rd quartiles (Q1, Q3), as appropriate. Data normality was checked after examining the histograms and Q–Q plots. Absolute values and relative frequencies are presented for categorical variables. Protein intensity and IGF-1 quantification were log2-transformed and scaled.

A multivariable generalized linear mixed-effects model (GLMM) was used to explore pregnancy-associated cardiac RR by including the 3rd trimester of pregnancy and 6 months after delivery. The LVM (including all LVM measured in 3rd trimester and 6 months after delivery) was defined as the only outcome in all GLMM performed. The follow-up time (3rd trimester and 6 months after delivery) was taken as a covariate, allowing the longitudinal analysis of cardiac (reverse) remodeling (using the left ventricular mass as surrogate). The analysis of LVM progression during pregnancy and postpartum was adjusted for CV risk factors, age at recruitment moment, parity, weight gain until 3rd trimester and evaluation moments (3rd trimester and 6 months after delivery) due to their clinical relevance. Only adjusted statistically significant proteins with an absolute effect size (beta coefficient) higher than 6 standard deviations and IGF-1 quantification were considered for exploratory analysis. A threshold of 6 standard deviations emerged from data-driven exploration of the volcano plot. This methodological approach prioritised minimising false positives given the sample size limitations and maximising the signal/noise ratio often present in proteomics analysis. *P* < 0.05 was deemed significant.

## Results

### Baseline demographic and clinical characterization

We included 59 participants that had paired urine samples at 32 [31; 34] weeks of gestation and 6 [6;7] months after delivery, with a median age of 34 [32; 37] years-old (50.8% nulliparous). The demographic and clinical characteristics of participants are shown in Table 1. Twenty-seven (45.8%) women were diagnosed with at least one CV risk factor prior to or during gestation (Table 1). The study groups were matched for smoking habits (*p* = 0.477), parity (*p* = 0.604) and obstetric outcomes (type of delivery, *p* = 0.279; gestational age at delivery, *p* = 0.291) (Table 1). In addition, women with CV risk factors were older (36 [33; 39] years versus 34 [30; 36] years, *p* = 0.049) than healthy participants. Aspirin was the only drug prescribed to 4 healthy pregnant women. Other pharmacological classes were prescribed exclusively to the participants in the HDO group. Regarding vascular reverse remodeling, a significant increase in PWV and, consequently, mean arterial pressure was noticed from 3rd trimester to 6 months after delivery in both study groups (Table 2). Women in the HDO group had higher blood pressure and arterial stiffness in both evaluation moments (Table 2). Three pregnancies developed pre-eclampsia (two hypertensive and one without any CV risk factor), and one from healthy group developed cholestasis of pregnancy. Two experienced other peripartum complications (arrhythmia was documented by one participant of the healthy group, and uncontrolled arterial hypertension was reported in hypertensive woman).

**Table 1:** Demographic and clinical baseline characteristics.

	Healthy group n = 32	HDO group n = 27	*p* value
Age, years	34 [30;36]	36 [33;39]	**0.049**
Height, meters	1.64 [1.60;1.69]	1.66 [1.60;1.70]	0.492
Pre-pregnancy body mass index, kg/m^2^	22.3 [20.8;24.3]	27.2 [22.2;31.7]	**0.001**
Cardiovascular risk factors			
Arterial hypertension n (%)	n/a	16 (59.3)	n/a
Obesity n (%)	n/a	11 (40.7)	n/a
Gestational diabetes n (%)	n/a	12 (44.4)	n/a
Type 2 Diabetes n (%)	n/a	1 (3.7)	n/a
Pharmacological therapy during pregnancy			
Aspirin	4 (12.5)	14 (51.9)	**0.002**
Metformin	0 (0.0)	11 (20.0)	** < 0.001**
Insulin	0 (0.0)	10 (18.2)	** < 0.001**
Beta-blockers	0 (0.0)	2 (7.4)	0.205
Nifedipine	0 (0.0)	1 (3.7)	0.458
Smoking habits			
Non-smoker n (%)	18 (56.3)	20 (74.1)	0.477
Smoker n (%)	3 (9.4)	2 (7.4)	
Stopped only during pregnancy n (%)	1 (3.1)	1 (3.7)	
Stopped at pregnancy beginning n (%)	2 (6.3)	2 (7.4)	
Ex-smoker n (%)	8 (25.0)	2 (7.4)	
Primiparous women n (%)	15 (46.9)	15 (55.6)	0.604
Pregnancy weeks at delivery	39 [31;33]	39 [38;40]	0.291
Type of delivery			
Vaginal delivery n (%)	19 (59.4)	20 (74.1)	0.279
Caesarean delivery n (%)	13 (40.6)	7 (25.9)	
Evaluation moment			
Pregnancy, weeks	31 [32;39]	32 [32;34]	0.146
Postpartum, months	7 [6;8]	6 [6;7]	**0.033**

Values expressed by median [interquartile range].

Significant values are in bold.

**Table 2:** Physical, vascular and echocardiographic assessment.

Group	Healthy group			HDO group			Healthy group versus HDO group			Healthy group	HDO group
Parameter/moment	3rd trimester	6 months postpartum	Variation	3rd trimester	6 months Postpartum	Variation	3^rd^ trimester *p* value	6 months postpartum *p* value	Variation *p* value	3rd trimester versus 6 months postpartum *p* value	3^rd^ trimester versus 6 months postpartum *p* value
Physical assessment											
Maternal body weight, kg	70 [67;81]	61 [57;69]	− 10 [− 11; − 8]	82 [73;95]	76 [64;89]	− 8 [− 10; − 3.8]	**0.004**	**0.003**	**0.043**	** < 0.001**	** < 0.001**
Weight variation, kg	11 [8;14]	− 10 [− 11; − 8]	− 20 [− 25; − 17]	9 [7;12.5]	− 8 [− 9.5; − 3.8]	− 15 [− 21; − 13]	0.089	**0.043**	**0.036**	** < 0.001**	** < 0.001**
Vascular assessment											
Systolic blood pressure, mmHg	110 [104;120]	110 [102;118]	0 [− 10; 9]	110 [110;128]	120 [114;140]	10 [0; 15]	0.160	** < 0.001**	**0.022**	0.753	**0.009**
Diastolic blood pressure, mmHg	65 [60;70]	76 [70;80]	10 [0; 17]	80 [70;80]	82 [80;93]	10 [0; 20]	** < 0.001**	** < 0.001**	0.565	** < 0.001**	** < 0.001**
Mean arterial pressure, mmHg	80 [76;86]	88 [84;91]	7 [− 2; 13]	90 [82;97]	95 [90;108]	7 [3; 18]	**0.003**	** < 0.001**	0.188	** < 0.001**	** < 0.001**
Pulse wave velocity, cm/s	6.0 [5.3;6.3]	6.7 [6.2;7.3]	0.7 [0.5; 1.3]	6.7 [5.9;7.3]	7.2 [6.7;8.0]	0.7 [0.3; 1.2]	** < 0.001**	**0.021**	0.451	** < 0.001**	** < 0.001**
Echocardiographic assessment											
Heart rate, beats/min	78 [70;85]	62 [58;67]	− 14 [− 21; − 9]	82 [72;86]	66 [64;72]	− 12 [− 22; − 6]	0.364	0.027	0.690	** < 0.001**	** < 0.001**
LVEF, %	60 [57;62]	62 [60;65]	3.0 [0.6; 7.8]	61 [58;62]	61 [58;63]	0.4 [− 2.0; 4.0]	0.622	0.163	**0.032**	**0.008**	0.493
Global longitudinal strain, %	− 23.3 [− 26.2; − 21.3]	− 23.7 [− 25.4; − 21.5]	− 1.2 [− 2.7; 3.7]	− 21.0 [− 22.1; − 20.1]	− 22.3 [− 23.5; − 20.4]	− 1.6 [− 4.4; 1.3]	**0.019**	0.073	0.245	0.753	0.140
Mitral inflow parameters											
E, cm/s	88 [77;96]	82 [72;90]	− 7 [− 17; 2]	92 [78;101]	80 [72;88]	− 2 [− 19; 3]	0.867	0.690	0.606	**0.009**	0.061
A, cm/s	59 [52;74]	48 [42;54]	− 14 [− 21; − 6]	66 [59;74]	52 [44;71]	− 13 [− 18; − 7]	0.171	0.097	0.600	** < 0.001**	** < 0.001**
E/A	1.43 [1.26;1.70]	1.69 [1.43;1.94]	0.19 [0.01; 0.51]	1.33 [1.09;1.53]	1.47 [1.31;1.66]	0.22 [− 0.05; 0.35]	0.161	0.062	0.542	** < 0.001**	**0.007**
Pulmonary vein inflow parameters											
S, cm/s	55 [48;59]	48 [446;56]	− 4 [− 12; 0]	56 [50;67]	51 [45;60]	− 8 [− 12; 2]	0.522	0.089	0.777	**0.028**	0.076
D, cm/s	50 [47;57]	52 [41;60]	− 1 [− 10; 5]	52 [43;58]	49 [41;53]	− 6 [− 11; 3]	0.089	0.402	0.349	0.637	0.088
S/D	0.93 [0.83;1.21]	1.08 [0.78;1.27]	− 0.11 [− 0.34; 0.11]	1.17 [0.99;1.25]	1.15 [1.06;1.28]	0.04 [− 0.11; 0.14]	0.332	0.152	0.325	0.122	0.557
TDI mitral annulus											
Septal											
e′ sept, cm/s	10.8 [9.5;12.6]	12.2 [10.8;13.3]	1.1 [− 0.1; 2.0]	10.1 [8.6;11.2]	10.8 [9.6;11.9]	1.1 [− 0.2;1.8]	0.103	**0.006**	0.508	**0.001**	0.088
Lateral											
e′ lat, cm/s	17.2 [15.2;19.3]	17.9 [16.0;19.8]	1.3 [− 1.1; 2.6]	15.5 [12.6;18.3]	15.3 [14.4;17.1]	− 0.6 [− 1.1; 1.6]	**0.054**	**0.005**	0.219	0.079	0.732
E/e′ average (E/e′)	6.3 [5.6;7.4]	5.6 [4.9;6.0]	− 1.1 [− 1.7; − 0.1]	7.0 [5.7;8.2]	6.1 [5.4;7.4]	− 0.6 [− 1.4; − 0.1]	0.122	**0.008**	0.552	** < 0.001**	**0.008**
Stroke volume, mL	62 [53;69]	60 [51;65]	− 3 [− 11;3]	60 [53;65]	60 [46;68]	− 2 [− 12; 2]	0.486	0.847	0.895	0.131	0.166
Cardiac output, L/min	5.0 [4.2;5.5]	3.7 [3.2;4.1]	− 1 [− 2; − 1]	4.9 [4.1;5.5]	3.8 [2.8;4.5]	− 1 [− 2; − 1]	0.865	0.905	0.841	** < 0.001**	** < 0.001**
Relative wall thickness	0.34 [0.31;0.38]	0.31 [0.29;0.33]	− 0.03 [− 0.05; 0.01]	0.37 [0.33;0.41]	0.34 [0.30;0.37]	− 0.05 [− 0.10; 0.01]	0.061	0.093	0.553	**0.007**	**0.017**
LV mass, g	129 [114;135]	99 [94;109]	− 22 [− 35; − 15]	128 [109;158]	112 [101;133]	− 12 [− 33; 3]	0.475	**0.014**	0.207	** < 0.001**	**0.003**
Maximum LA volume, mL	49 [45;53]	38 [31;43]	− 11 [− 18; − 6]	51 [47;56]	43 [37;51]	− 10 [− 14; − 5]	0.317	**0.045**	0.258	** < 0.001**	**0.006**
LV end-diastolic volume, mL	94 [82;100]	78 [71;86]	− 14 [− 18; − 7]	101 [85;108]	88 [75;100]	− 7 [− 20; 1]	0.209	**0.029**	0.128	** < 0.001**	**0.006**

Values expressed by median [interquartile range].

Significant values are in bold.

### Characterization of cardiac reverse remodeling

Both groups similarly decreased heart rate and cardiac output from the 3^rd^ trimester to 6 months after delivery (Table 2). Pregnancy impaired diastolic function in both groups, as assessed by a significant decrease of E/A and an increase in E/e′ and left atrial volume (LAV) compared to the postpartum period (Table 2). At postpartum, participants showed a significant reduction in LVM, volume and relative wall thickness, irrespective of the presence of CV risk factors (Table 2).

Interestingly, global longitudinal strain was the only parameter significantly reduced in the 3rd trimester of HDO pregnant women (− 23.3[− 26.2; − 21.3]% versus − 21.0[− 22.1; − 20.1]%, *p* = 0.019). After delivery, the cardiac anatomic and functional normalization was much less noticeable in the HDO group, which maintained values significantly higher than those in the healthy group (Table 2). The diastolic changes observed in the HDO group become significantly different from those in the healthy group at 6 months postpartum (E/e′, 6.1[5.4;7.4] versus 5.6[4.9;6.0], *p* = 0.008; LAV, 43[37;51]mL versus 38[31;43]mL, *p* = 0.045).

### Identification of proteins in urine sample

Over 2700 proteins were identified in urine samples through LC–MS/MS, after excluding reverse sequences, potential contaminants and proteins only identified by site. To explore the predictive power of the urinary proteins, we only considered proteins with two or more peptides identified and proteins with less than 20% of missing values, resulting in a selection of 324 proteins (Supplemental Table 2; Supplemental file 1).

### Urinary proteins showing an association with cardiac reverse remodeling

**Figure 1:**
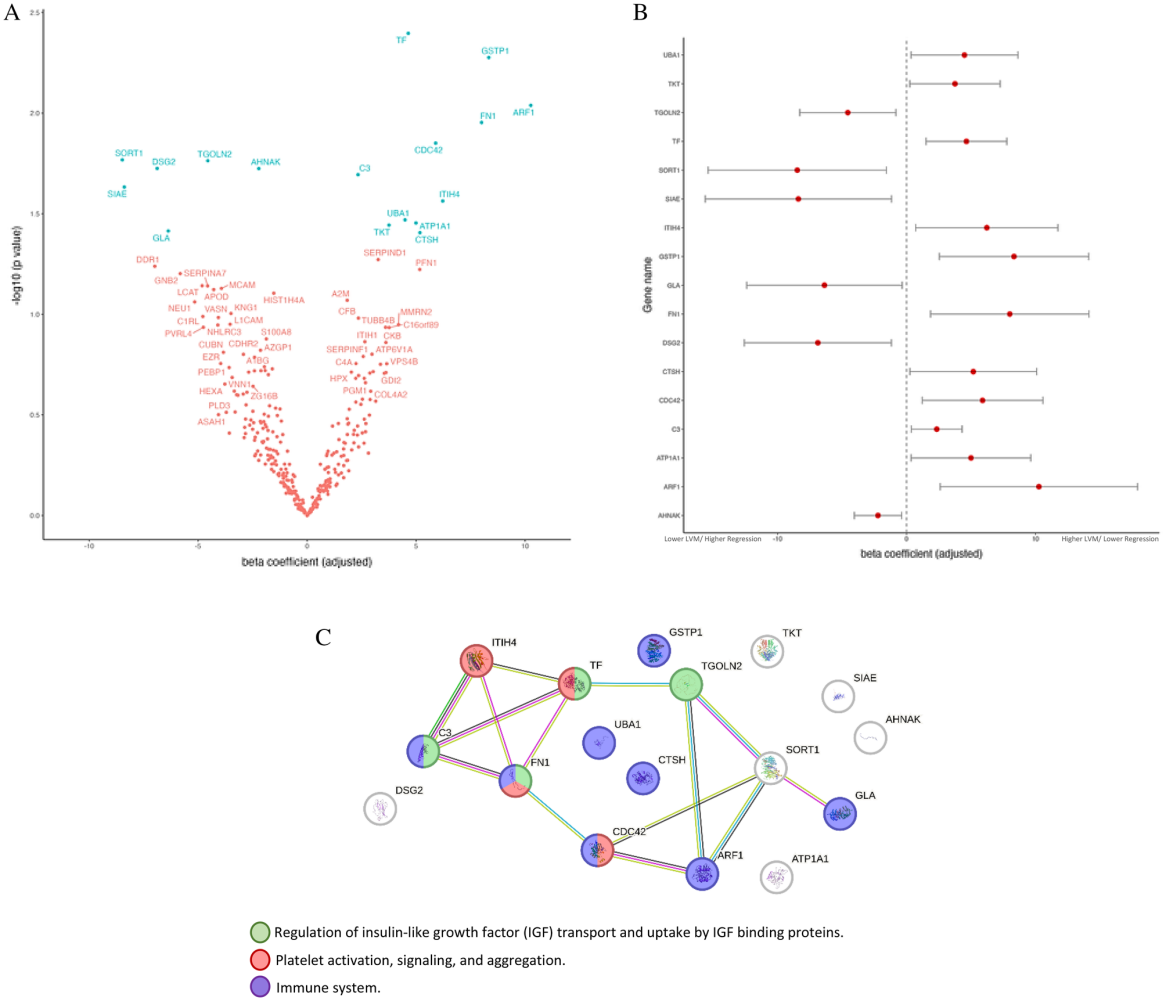
(**A**) Volcano Plot from multivariable generalized linear mixed-effects models (adjusted to cardiovascular risk factors, age at recruitment moment, parity, weight gain until 3rd trimester, and evaluation moments) showing the distribution of the proteins according to their effect (beta coefficient) and significance level (− log_10_(*p*-value)) regarding LVM regression. Proteins are identified with the respective gene name. Red labels are non-significant associations whereas blue labels signify significant associations. (**B**) Urine proteins showing a significant association with LVM, according to the GLMM. (**C**) STRING Protein–Protein interaction network of the 17 most relevant proteins associated with postpartum LVM, according to a GLMM. Each node represents a protein, identified with the respective gene name. The most relevant biological processes found through gene ontology enrichment analysis are depicted through node colors. Each type of protein interaction is represented by a different edge color (cyan: known interaction from curated databases, pink: experimentally determined; yellow: text mining; black: co-expression; green: predicted interaction from gene neighborhood).

The LVM was defined as the outcome in a multivariable generalized linear mixed-effects model (GLMM) to identify urinary proteins associated with postpartum cardiac RR. In the pre-specified GLMM containing only clinical variables, the presence of at least one CV risk factor (β: 1.4, 95% CI [3.30; 24.0], *p* = 0.011) was associated with low LVM regression after delivery (Table 3). Upon addition of each one of 324 proteins on TOP to the pre-specified model, seventeen urinary proteins were associated with LVM (Fig. 1A and B). According to STRING network configuration, many of these proteins interact amongst them, participating in common pathways. This includes the regulation of insulin-like growth factor (IGF) transport and uptake by IGF binding proteins (involving Complement C3 [C3], Fibronectin [FN1], Serotransferrin [TF] and Trans-Golgi network integral membrane protein 2 [TGOLN2]); platelet activation, signaling and aggregation (including Inter-alpha-trypsin inhibitor heavy chain H4 [ITIH4], Serotransferrin [TF], Fibronectin [FN1] and Cell division control protein 42 homolog [CDC42]) and immune system (namely, Complement C3 [C3], Fibronectin [FN1], Cell division control protein 42 homolog [CDC42], ADP-ribosylation factor 1 [ARF1], Alpha-galactosidase A [GLA], Ubiquitin-like modifier-activating enzyme 1 [UBA 1], Pro-cathepsin H [CTSH] and Glutathione S-transferase P [GSTP1]) (Fig. 1C).

**Table 3:** Multivariable generalized linear mixed models for LVM.

Parameter	β, 95% CI	*p* value
**Pre-specified clinical model**		
Follow-up time	− 20.00 [− 26.00; − 15.00]	** < 0.001**
Age	− 0.79 [− 2.10;0.50]	0.200
Cardiovascular risk group	14.00 [3.30;24.00]	**0.011**
Nulliparous	− 5.70 [− 17.00;5.10]	0.300
Weight gain during pregnancy	0.28 [− 0.75;1.30]	0.600
**Pre-specified clinical model with inclusion of IGF-1 quantification**		
Follow-up time	− 13.00 [− 22.00; − 4.60]	**0.003**
Age	− 0.82 [− 2.10;0.50]	0.200
Cardiovascular risk group	10.00 [− 0.47;21.00]	0.060
Nulliparous	− 6.90 [− 18.00, 4.10]	0.200
Weight gain during pregnancy	0.09 [− 0.93;1.10]	0.900
Log-transformed insulin-like growth factor-1	6.10 [0.18;12.00]	**0.044**

Significant values are in bold.

We then evaluated the added value of the proteins to the clinical model to predict LVM regression. For this, we only considered relevant urinary proteins with an absolute magnitude (expressed by the beta coefficient) higher than 6 (Inter-alpha-trypsin inhibitor heavy chain H4 [ITIH4], Glutathione S-transferase P [GSTP1], Fibronectin [FN1], ADP-ribosylation factor 1 [ARF1], Alpha-galactosidase A [GLA], Sialate O-acetylesterase [SIAE], Sortilin [SORT1], Desmoglein-2 [DSG2]). Nevertheless, the model performance improved significantly (*p* < 0.001, Table 4). Of note, the combination of the 8 proteins, or the single addition of the proteins GSTP1, SORT1, or DSG2 increased model performance regarding LVM regression (lowest AIC [Akaike Information Criterion] and higher R^2^).

**Table 4:** Performance of multivariable generalized linear mixed-effects model.

	AIC	R^2^ (cond)	R^2^ (marg.)	RMSE
Pre-specified clinical variables multivariable GLMM	1032.8	0.63	0.24	12.087
Pre-specified clinical variables multivariable GLMM + GSTP1	861.9	0.70	0.25	10.015
Pre-specified clinical variables multivariable GLMM + ARF1	864.6	0.68	0.25	10.622
Pre-specified clinical variables multivariable GLMM + FN1	1024.3	0.67	0.28	11.368
Pre-specified clinical variables multivariable GLMM + SORT1	870.1	**0.72**	0.26	9.829
Pre-specified clinical variables multivariable GLMM + SIAE	889.8	0.61	0.29	12.535
Pre-specified clinical variables multivariable GLMM + GLA	959.2	0.62	0.28	12.226
Pre-specified clinical variables multivariable GLMM + ITIH4	1027.8	0.61	0.27	12.309
Pre-specified clinical variables multivariable GLMM + DSG2	837.2	**0.70**	0.25	10.482
Pre-specified clinical variables multivariable GLMM + panel of last 8 urinary proteins	621.0	**0.70**	0.28	9.638

AIC, akaike information criterion; ARF1, ADP-ribosylation factor 1; cond, conditional; DSG2, desmoglein-2; FN1, fibronectin; GLA, alpha-galactosidase A; GSTP1, glutathione S-transferase P1; ITIH4, inter-α-trypsin inhibitor heavy chain 4; marg., marginal; R^2^, coefficient of determination; RMSE, root mean square error; SORT1, sortilin; SIAE, sialate O-acetylesterase. The pre-specified clinical variables included in multivariable generalized linear mixed-effects model (GLMM) were: CV risk factors, age at recruitment moment, parity, weight gain until 3rd trimester and evaluation moments (3rd trimester and 6 months after delivery) due to their clinical relevance. The LVM was defined as the outcome in all GLMMs.

### Insulin-like growth factor-1 showing an association with cardiac reverse remodeling

Insulin-like growth factor-1 (IGF-1) is involved in the pathway of the regulation of IGF transport and uptake by IGF-binding proteins. In our cohort, increased urinary levels of IGF-1, quantified through ELISA Assay Kit were associated with low LVM regression after delivery (Table 3).

## Discussion

This work is the first exploratory prospective cohort study that included healthy pregnant women and with CV risk factors to identify urinary proteins associated with cardiac RR, as assessed through LVM variation, from the 3rd trimester to 6 months after delivery.

Our cohort showed a significant regression of LVM and volume, and LAV, associated with an improvement of diastolic function (E/e′) from the 3rd trimester to 6 months after delivery, confirming previous reports^2,3^. Although pregnant women with and without CV risk factors have similar LVM in the 3rd trimester, pregnant women with CV risk factors showed lower LVM regression, that is, incomplete cardiac RR at 6 months postpartum compared to healthier ones. The same holds true for diastolic function in the CVR factors group. Indeed, pregnancy seems to mask the diastolic changes observed in the HDO group, as these differences only become evident and significantly different from those in the healthy group at 6 months postpartum. The presence of at least one CV risk factor (arterial hypertension, obesity, gestational diabetes or type-II diabetes) was associated with worse LVM regression. Additionally, women with these cardiometabolic comorbidities had higher postpartum cardiac volumes and LV filling pressure than those in the healthy group, as confirmed by previous studies focusing on hypertensive diseases of pregnancy^13–15^. In addition, both study groups showed an increase in arterial stiffness between evaluation moments, being significantly elevated in women with CV risk factors, which is in line with previous reports^16–19^.

To identify proteins associated with higher cardiac RR, we profiled the urine proteome before and after delivery in pregnant women with and without CV risk factors. We then assessed whether these proteins could independently predict postpartum LVM. Alpha-galactosidase A (GLA), sialate O-acetylesterase (SIAE), trans-golgi network integral membrane protein 2 (TGOLN2), sortilin (SORT1), desmoglein-2 (DSG2) and neuroblast differentiation-associated protein (AHNAK) were found to be potential indicators of a higher LVM regression (β < 0). In contrast, serotransferrin (TF), inter-α-trypsin inhibitor heavy chain 4 (ITIH4), complement C3 (C3), transketolase (TKT), fibronectin (FN1), ubiquitin-like modifier-activating enzyme 1 (UBA1), cell division control protein 42 homolog (CDC42), glutathione s-transferase P1 (GSTP1), sodium/potassium-transporting ATPase subunit alpha-1 (ATP1A1), ADP-ribosylation factor 1 (ARF1) and pro-cathepsin H (CTSH) were associated with a lower LVM regression after delivery (β > 0).

Many putative proteins associated with LVM were found to be functionally integrated with biological pathways, such as the *regulation of insulin-like growth factor (IGF) transport and uptake by IGF-binding proteins*; *platelet activation, signaling and aggregation* and *immune system activity*. These signaling pathways evidence a complex systemic interplay between organs during pregnancy, including women’s response to partum-induced cardiac RR.

For instance, insulin-like growth factor-I (IGF-1) can bind to IGF-1 receptors and activate the Akt pathway^20^, thereby promoting growth. IGF-1/PI3K/Akt pathway is involved in LV hypertrophy^21^, including pregnancy-induced cardiac hypertrophy^22^. Thus, IGF bioavailability regulation, through binding to IGF-binding proteins, may impact IGF-induced pro-hypertrophic cardiac activity. Interestingly, three out of four proteins associated with IGF transport and uptake (TF, C3 and FN1) were associated with worse LVM regression, which suggests that IGF signaling blockage can be a protective mechanism against cardiac hypertrophy in the pregnancy setting. Corroborating it, the IGF-1 levels quantified in urine samples of our cohort were associated with low LVM regression after delivery. Among the proteins mapped to this pathway, we reported for the first time that FN1 is associated with low LVM regression. Indeed, FN1 has been correlated with cardiac remodeling induced by volume overload and arterial hypertension^23^. Fibronectin accelerates pathological hypertrophy, leading to the expression of fetal genes, such as ANP, BNP, SkA, and MHC^23,24^. Thereby, FN1 is a potential target for modification of hypertrophic phenotype^24^. Intimately correlated with an increase in FN1 is the ubiquitin–proteasome pathway, responsible for the degradation of most intracellular proteins in the heart^25,26^. For instance, UBA1, a protein involved in the initial step of ubiquitylation^25^ whose role in cardiac remodeling and RR regulation remains unclear, was associated with lower LVM regression in our cohort. In our previous study, ubiquitin correlated positively with interventricular septum and posterior wall thickness (surrogates of cardiac hypertrophy) in patients with aortic stenosis undergoing valve replacement^27^. Moreover, the inhibition of UBA1 by PYR-41 was linked to a significant decrease in blood pressure, cardiac hypertrophy, fibrosis, oxidative stress and inflammation and improved angiotensin II-induced cardiac contractile dysfunction^25^.

According to STRING’s functional analysis, some proteins associated with postpartum LVM, namely FN1, TF, ITIH4, and CDC42, are involved in *platelet activation, signaling and aggregation*. Platelets are actively involved in regulating vascular tone and inflammation and participate in the immune system, showing an important role in hemostasis, thrombosis and the pathophysiology of uteroplacental diseases^28,29^. Increased platelet aggregation accompanied by a reduction of circulating platelets has been reported throughout pregnancy^29–31^. The decrease in platelet count may result from physiologic hemodilution or from platelet aggregation. In this case, it may lead to uteroplacental diseases such as recurrent miscarriage and pre-eclampsia^29^. The low number of preeclampsia cases present in our cohort may be explained since approximately half of the participants with CV risk factors were taking acetylsalicylic acid, a platelet aggregation inhibitor, according to their risk for preeclampsia estimated by *The Fetal Medicine Foundation* algorithm. Indeed, the American College of Obstetricians and Gynecologists (ACOG) recommended low-dose aspirin prophylaxis for women with a high risk of pre-eclampsia^32^. Serotransferrin (TF) is involved in iron transfer from the placenta to the fetus through the transferrin receptor in the syncytiotrophoblast membrane. Therefore, it rises towards the end of pregnancy to meet the elevated iron requirements for fetus development^33^. Our findings suggest that CV risk factors mediate the positive association between TF and LVM (i.e., higher gestational TF is associated with worse cardiac mass regression), as observed in our study and supported by studies showing TF overexpression in women with CV risk factors^34^. Regarding *platelet activation, signaling and aggregation*, ITIH4 belongs to the ITIH family and stabilizes the extracellular matrix^35^. Therefore, high levels of ITIH proteins may have an inhibitory effect on the degradation of extracellular matrix, thereby maintaining anti-angiogenic^36^ and hypertrophy effects. This might explain its inverse association with LVM regression. CDC42 holds a critical role in cardiomyocyte proliferation, sarcomere organization and cell–cell adhesion^37^. Furthermore, CDC42 is a member of the Rho GTPase family and may regulate LV hypertrophy, directly controlling non-contractile actin and microtubules cytoskeleton formation through JNK anti-hypertrophic pathway^38^. However, CDC42 shows controversial results, having been described as either pro- or anti-cardiac hypertrophic signaling actions in response to physiological and pathological conditions, depending on the experimental setting^39,40^. In our cohort, which included diabetic and hypertensive women, CDC42 demonstrated a positive correlation with LVM. This observation is consistent with the hormone-induced proliferative state of pregnancy. Of note, if and how the regulation of platelet-mediated hemostasis through FN1/TF/ITITH4/CDC42 impacts cardiovascular remodeling during pregnancy remains elusive and deserves further scrutiny.

According to STRING, the *immune system* is also an important player among the proteins deemed to be more strongly associated with cardiac RR. All phases of pregnancy, from implantation to fetal growth and parturition, require a fine-tuning of immunological processes^41^, consistent with pregnancy’s cardiovascular adaptations. We found the complement system protein C3 to be positively correlated with LVM. Indeed, in coronary heart disease, C3 inhibition seems to restrain hypertrophy and improve cardiac function and survival^42^. In contrast, *Shahini *et al*.* showed that C3 inhibition did not affect the cardiac maladaptive remodeling in response to pressure overload^43^. Among the 8 proteins mapped to the immune system, GSTP1, an anti-inflammatory protein, was upregulated in the hypertrophied LV to balance elevated levels of reactive oxygen species in heart failure patients, being an independent predictor of adverse cardiac remodeling^44,45^.

The pre-specified clinical model to assess the probability of postpartum RR found that 8 proteins (ITIH4, GSTP1, FN1, ARF1, GLA, SIAE, SORT1, DSG2) could improve the model’s performance individually (GSTP1, DSG2 and SORT-1) or combined. Therefore, we developed the first model, including clinical variables and urinary proteins, to assess cardiac RR, through LVM quantification during and after pregnancy. Regarding the proteins relevant to improving the robustness of the GLM model, we found an inverse association between GLA, DSG2, SIAE and SORT1 expression and LVM. Curiously, despite most of these not being described previously in the context of cardiac remodeling or RR^46–49^, they do predict postpartum cardiac RR.

This highlights the relevance of the present study and the need to validate and unravel the involved molecular mechanisms and pathways. In this context, we desire to validate the eight proteins (ITIH4, GSTP1, FN1, ARF1, GLA, SIAE, SORT1, DSG2) in an independent cohort of pregnant women. Beyond the functional validation through IGF-1 in the present work, we want to explore the mechanisms of the most relevant biological pathways associated with incomplete cardiac RR using an already established model of pregnancy in Wistar rats^50^.

Pregnancy becomes an important window to predict and prevent future cardiovascular events and heart failure progression in women with cardiometabolic comorbidities^51^. The incomplete cardiac RR pattern that we showed in the present study has been reported in women with a history of pregnancy hypertensive diseases, who show a higher probability of developing concentric left ventricular (LV) remodeling accompanied by LV diastolic dysfunction, substrates for Heart Failure with Preserved Ejection Fraction (HFpEF) development later in life^51^. For this reason, we believe identifying potential urinary proteins (ITIH4, GSTP1, FN1, ARF1, GLA, SIAE, SORT1, DSG2) as biomarkers of incomplete postpartum RR will be promising for signaling women with a higher risk of developing HFpEF at the long-term. Identifying these women during pregnancy may promote a close medical follow-up after delivery and better pharmacological therapy optimisation, reducing HFpEF incidence. Regarding study limitations, COVID-19 pandemic strongly impacted the progression of this study by limiting participants’ recruitment and cardiovascular evaluation. This resulted in follow-up losses and, consequently, a reduced sample size, compromising the proteomics analysis according to the study group. This also precluded the comparison of cardiac RR and its association with a urinary proteomic profile according to each cardiovascular risk (chronic arterial hypertension versus obesity versus type 2 diabetes versus gestational hypertension versus gestational diabetes). The lack of adjustment for multiple testing performed in this study may also be considered a limitation. However, we used stringent effects size criteria to select the putative proteins to inform the extent of cardiac RR in the model. Furthermore, the participants were stratified at the recruitment moment according to their baseline conditions (presence or absence of cardiovascular risk factors, namely chronic hypertension, gestational hypertension, gestational diabetes and obesity), not taking into account potential subsequent crossovers (such as the diagnosis of pre-eclampsia at the end of pregnancy).

In conclusion, we found that cardiac RR was characterized by significant LV chamber regression and diastolic function improvement. However, pregnant women with CV risk factors revealed compromised LVM regression and diastolic function improvement at postpartum. The proteins GSTP1, ARF1, FN1, SORT1, SIAE, GLA, ITIH4 and DGS2 were independently associated with LVM regression, and all improved the performance of a model for predicting cardiac RR. Indeed, the proteomic profile of urine reflected the cardiac reverse remodeling process, endorsing urine protein analysis as an alternative or complementary approach to assess the risk of postpartum incomplete cardiac RR. In addition, the association between IGF and postpartum LVM regression was validated.

### Supplementary Information

Supplementary Tables.

Supplementary Information.

## Data Availability

The mass spectrometry proteomics data have been deposited to the PRIDE Archive (http://www.ebi.ac.uk/pride/archive/) via the PRIDE partner repository with the data set identifier PXD042655.

## Abbreviations

AHNAK: Neuroblast differentiation-associated protein
ARF1: ADP-ribosylation factor 1
ATP1A1: Sodium/potassium-transporting ATPase subunit alpha-1
CV: Cardiovascular
CDC42: Cell division control protein 42 homolog
C3: Complement C3
CTSH: Pro-cathepsin H
DM: Diabetes mellitus
DSG2: Desmoglein-2
FN1: Fibronectin
GLA: Alpha-galactosidase A
GLMM: Generalized linear mixed-effects model
GSTP1: Glutathione S-transferase P
HDO: Combination of hypertensive, diabetic and obese women
IGF: Insulin-like growth factor
IGF-1: Insulin-like growth factor-1
ITIH4: Inter-alpha-trypsin inhibitor heavy chain H4
LC–MS/MS: Liquid chromatography tandem mass spectrometry method
LA: Left atrium
LV: Left ventricle
LVM: Left ventricle mass
PWV: Pulse wave velocity
RR: Reverse remodeling
SIAE: Sialate O-acetylesterase
SORT1: Sortilin
TF: Serotransferrin
TGOLN2: Trans-golgi network integral membrane protein 2
TKT: Transketolase
3T: Third trimester
UBA 1: Ubiquitin-like modifier-activating enzyme 1
