# Supplementary material for: The extent of postpartum cardiac reverse remodeling is reflected in urine proteome
Source: Sci Rep. 2024 Jun 27;14:14815. doi: 10.1038/s41598-024-65612-1 (PMC11211500; doi:10.1038/s41598-024-65612-1)
Supplement: Supplementary file 1 — Supplementary Tables. [file 41598_2024_65612_MOESM1_ESM.pdf]

Supplemental Table 1: Echocardiographic equations applied in the present study.

**Echocardiographic Variables**	**Equations**	**Unit**
LVM	$0.8\times(1.04\times[(LVEDd+PWd+IVSd)^3–LVEDd^3]+0.6)$	g
RWT	$2\times PWd/LVEDd$	n/a
Ejection fraction	$LVIDd^3-LVIDs^3/LVIDd^3\times 100$	%
LV cardiac output	*LV stroke volume x Heart rate*	L/min
Mean arterial pressure	*(SBP + 2 x DBP)/3*	*mmHg*
E/e’	*E mitral inflow velocity / average of Early Diastolic Septal and Lateral mitral annular velocity*	n/a

Legend: CO – cardiac output; DBP – diastolic blood pressure; IVSd - interventricular septum thickness in diastole; LV – left ventricular; LVEDd - left ventricular end-diastolic diameter; LVIDd - left ventricular internal end-diastolic dimension; LVIDd - left ventricular internal end-systolic dimension; LVM – left ventricular mass; PWd - left ventricular posterior wall thickness in diastole; RWT – relative wall thickness; SBP – systolic blood pressure.

Supplemental Table 2: Urinary proteins identified through LC-MS/MS and included in pre-specified clinical variables multivariable GLMM for LVM.

Protein ID	Gene Name	N	Beta	95% CI[1]	p-value
P07911	UMOD	118	-1.7	-4.9, 1.5	0.3
P0DOY2	IGLC2	102	-1.6	-5.1, 2.0	0.4
P01619	IGKV3-20	116	0.79	-3.3, 4.9	0.7
P41222	PTGDS	117	-0.70	-3.9, 2.5	0.7
P0DJD8	PGA3	105	-2.0	-4.9, 0.94	0.2
P01857	IGHG1	117	2.1	-2.8, 7.1	0.4
P01009	SERPINA1	118	2.6	-1.5, 6.7	0.2
P01876	IGHA1	117	-1.5	-6.7, 3.6	0.6
P05154	SERPINA5	118	0.82	-2.2, 3.8	0.6
P62805	HIST1H4A	99	-1.5	-3.2, 0.18	0.079
P01042	KNG1	118	-3.5	-7.7, 0.67	0.10
P01011	SERPINA3	118	0.32	-3.4, 4.0	0.9
P05155	SERPING1	118	-1.9	-6.9, 3.1	0.5
P10909	CLU	118	-1.3	-5.2, 2.5	0.5
P08571	CD14	118	0.38	-3.5, 4.2	0.8
B9A064	IGLL5	117	-0.81	-3.5, 1.9	0.6
P04083	ANXA1	118	-1.6	-4.0, 0.79	0.2
Q08380	LGALS3BP	118	-0.24	-4.7, 4.2	>0.9
Q01469	FABP5	117	-0.58	-3.0, 1.9	0.6
P04746	AMY2A	117	-1.7	-6.3, 2.9	0.5
Q9UBC9	SPRR3	114	-1.2	-3.6, 1.1	0.3
P01833	PIGR	117	-1.3	-6.7, 4.1	0.6
Q6EMK4	VASN	116	-4.1	-9.0, 0.85	0.10
P02787	TF	118	4.6	1.5, 7.8	**0.004**
P29508	SERPINB3	118	0.90	-1.8, 3.6	0.5
Q14624	ITIH4	118	6.2	0.71, 12	**0.027**
P02760	AMBP	117	0.04	-4.5, 4.5	>0.9
P01133	EGF	118	-2.5	-7.6, 2.7	0.3
P07355	ANXA2	118	-1.4	-4.2, 1.3	0.3
P60709	ACTB	118	2.9	-2.8, 8.5	0.3

Protein ID	Gene Name	N	Beta	95% CI[1]	p-value
P54802	NAGLU	118	-1.5	-7.4, 4.3	0.6
P80188	LCN2	111	-0.64	-3.2, 2.0	0.6
P10253	GAA	118	-1.3	-6.4, 3.8	0.6
P01019	AGT	116	0.91	-2.3, 4.2	0.6
P24855	DNASE1	116	1.1	-3.7, 5.8	0.7
Q9UNN8	PROCR	110	-1.1	-5.3, 3.2	0.6
Q13510	ASAH1	118	-4.1	-12, 3.9	0.3
P07339	CTSD	118	-1.1	-6.4, 4.2	0.7
P11117	ACP2	117	-0.40	-6.4, 5.6	0.9
P10451	SPP1	115	-0.60	-4.4, 3.2	0.8
P02647	APOA1	115	0.61	-1.4, 2.7	0.6
P05109	S100A8	110	-1.9	-4.3, 0.58	0.13
P05090	APOD	110	-4.3	-9.0, 0.45	0.075
P01871	IGHM	116	-0.64	-4.1, 2.9	0.7
Q9NQ84	GPRC5C	117	0.48	-4.0, 5.0	0.8
O00560	SDCBP	118	0.59	-3.0, 4.2	0.7
Q96DA0	ZG16B	113	-2.5	-6.5, 1.6	0.2
P15144	ANPEP	118	-0.12	-6.7, 6.4	>0.9
P16278	GLB1	118	-2.5	-8.0, 3.0	0.4
P08185	SERPINA6	116	1.7	-2.7, 6.2	0.4
P00738	HP	107	-0.20	-2.2, 1.8	0.8
P19835	CEL	118	-0.24	-4.9, 4.4	>0.9
Q12805	EFEMP1	117	0.41	-4.5, 5.3	0.9
P30086	PEBP1	115	-3.6	-8.9, 1.7	0.2
P07195	LDHB	118	-0.80	-6.4, 4.7	0.8
Q8NFZ8	CADM4	111	-2.0	-7.0, 3.0	0.4
P15586	GNS	118	-2.5	-7.3, 2.3	0.3
Q9NZZ3	CHMP5	109	-1.8	-6.6, 3.0	0.5
P05543	SERPINA7	117	-4.6	-9.6, 0.42	0.072
P02790	HPX	111	2.0	-1.0, 5.1	0.2
P02671	FGA	108	0.96	-1.7, 3.7	0.5

Protein ID	Gene Name	N	Beta	95% CI[1]	p-value
P00450	CP	118	-1.0	-4.5, 2.4	0.6
P22891	PROZ	116	0.47	-3.6, 4.6	0.8
P05062	ALDOB	115	-0.10	-4.5, 4.3	>0.9
P16444	DPEP1	118	-0.21	-5.3, 4.9	>0.9
P53990	IST1	117	0.86	-4.0, 5.7	0.7
P25311	AZGP1	114	-2.1	-5.1, 0.79	0.2
P30740	SERPINB1	115	-0.90	-3.4, 1.6	0.5
P12109	COL6A1	117	-0.26	-5.9, 5.4	>0.9
Q7LBR1	CHMP1B	108	-1.8	-5.8, 2.2	0.4
Q9Y646	CPQ	118	-1.0	-6.3, 4.2	0.7
P06733	ENO1	118	1.8	-2.4, 6.1	0.4
P15309	ACPP	115	0.98	-2.8, 4.8	0.6
P02649	APOE	117	-0.57	-5.8, 4.6	0.8
O43633	CHMP2A	111	0.69	-3.5, 4.9	0.7
P12830	CDH1	114	-1.8	-5.3, 1.8	0.3
O60494	CUBN	118	-3.8	-9.2, 1.5	0.2
P04217	A1BG	105	-2.4	-5.8, 1.0	0.2
Q93088	BHMT	115	-0.98	-4.5, 2.6	0.6
P01024	C3	118	2.3	0.37, 4.3	**0.020**
P19440	GGT1	116	-0.19	-5.8, 5.4	>0.9
P08294	SOD3	96	-0.34	-5.3, 4.6	0.9
P63104	YWHAZ	116	0.89	-4.2, 6.0	0.7
P31949	S100A11	98	-1.9	-5.9, 2.2	0.4
Q14019	COTL1	94	-0.53	-3.9, 2.8	0.8
P09210	GSTA2	95	-1.4	-5.4, 2.6	0.5
P04004	VTN	114	0.92	-1.7, 3.6	0.5
Q969P0	IGSF8	117	2.2	-2.7, 7.0	0.4
P33908	MAN1A1	115	1.9	-1.7, 5.6	0.3
P04075	ALDOA	118	-2.0	-6.5, 2.4	0.4
P39059	COL15A1	113	-2.0	-6.1, 2.1	0.3
Q16769	QPCT	100	0.30	-5.2, 5.8	>0.9

Protein ID	Gene Name	N	Beta	95% CI[1]	p-value
Q9NZP8	C1RL	113	-4.8	-11, 0.98	0.10
Q16270	IGFBP7	97	1.2	-2.3, 4.8	0.5
P14618	PKM	117	-1.5	-6.1, 3.2	0.5
Q01459	CTBS	112	-2.7	-6.7, 1.4	0.2
P00918	CA2	116	-0.27	-5.6, 5.1	>0.9
P98164	LRP2	118	-1.1	-6.6, 4.4	0.7
P50995	ANXA11	115	1.5	-2.1, 5.0	0.4
P0C0L5	C4B	118	2.4	-1.3, 6.0	0.2
Q9UBX5	FBLN5	117	1.9	-4.0, 7.7	0.5
P01008	SERPINC1	116	0.04	-3.5, 3.6	>0.9
P05156	CFI	113	0.27	-4.4, 4.9	>0.9
O95336	PGLS	116	-0.14	-5.1, 4.8	>0.9
P62879	GNB2	111	-5.8	-12, 0.32	0.063
P15941	MUC1	111	-2.6	-7.9, 2.7	0.3
P02750	LRG1	107	-1.2	-4.3, 1.8	0.4
P26038	MSN	118	0.84	-4.3, 5.9	0.7
O75882	ATRN	118	-1.7	-6.8, 3.5	0.5
P27105	STOM	116	0.18	-6.5, 6.8	>0.9
P00338	LDHA	113	-0.30	-5.5, 4.9	>0.9
P27487	DPP4	118	0.00	-6.0, 6.0	>0.9
P22792	CPN2	112	-0.86	-6.1, 4.4	0.7
P08758	ANXA5	115	0.86	-3.3, 5.0	0.7
P98160	HSPG2	118	1.1	-4.1, 6.2	0.7
P07737	PFN1	97	5.2	-0.22, 11	0.060
Q9UHL4	DPP7	113	-0.75	-5.5, 4.0	0.8
P12429	ANXA3	109	-1.2	-3.6, 1.3	0.3
Q07075	ENPEP	117	-0.29	-4.1, 3.5	0.9
A8K2U0	A2ML1	116	-0.18	-2.3, 1.9	0.9
P29622	SERPINA4	118	0.18	-4.4, 4.8	>0.9
Q92820	GGH	108	-1.6	-7.1, 3.8	0.6
Q6UX06	OLFM4	116	-0.94	-6.1, 4.2	0.7

Protein ID	Gene Name	N	Beta	95% CI[1]	p-value
P00558	PGK1	116	2.2	-1.8, 6.3	0.3
P09467	FBP1	113	0.04	-4.1, 4.2	>0.9
P13473	LAMP2	105	0.10	-3.8, 4.0	>0.9
Q8WUM4	PDCD6IP	118	-0.49	-4.8, 3.8	0.8
P60174	TPI1	115	2.6	-2.7, 8.0	0.3
Q06830	PRDX1	106	-2.4	-8.4, 3.5	0.4
P78324	SIRPA	115	-2.0	-5.8, 1.9	0.3
P43251	BTD	115	2.6	-4.0, 9.2	0.4
Q5VW32	BROX	112	-1.3	-6.7, 4.1	0.6
P23284	PPIB	106	0.12	-6.1, 6.3	>0.9
Q9NP79	VTA1	107	2.3	-3.1, 7.7	0.4
P08236	GUSB	109	-2.0	-7.2, 3.1	0.4
P08473	MME	118	1.6	-4.6, 7.7	0.6
P01023	A2M	112	1.8	-0.26, 3.9	0.085
Q9H3G5	CPVL	113	1.7	-2.9, 6.2	0.5
P52758	HRSP12	100	-1.1	-5.9, 3.7	0.7
P15289	ARSA	116	1.3	-3.4, 5.9	0.6
P07686	HEXB	118	-2.3	-8.6, 4.0	0.5
Q92692	PVRL2	110	-2.1	-8.4, 4.1	0.5
P04066	FUCA1	114	-2.4	-9.5, 4.7	0.5
Q9UKU9	ANGPTL2	111	0.95	-3.4, 5.3	0.7
P09525	ANXA4	117	-0.84	-6.2, 4.6	0.8
P68104	EEF1A1	102	1.0	-4.1, 6.2	0.7
P35858	IGFALS	114	0.77	-3.3, 4.8	0.7
P11597	CETP	105	-0.36	-3.8, 3.1	0.8
P36955	SERPINF1	118	2.6	-1.1, 6.2	0.2
P06865	HEXA	116	-3.8	-9.9, 2.3	0.2
P23528	CFL1	99	-0.96	-7.6, 5.7	0.8
P62834	RAP1A	104	-0.41	-5.7, 4.9	0.9
Q9H8L6	MMRN2	112	4.2	-1.0, 9.4	0.11
P53634	CTSC	113	-1.2	-7.1, 4.7	0.7

**Protein ID**	**Gene Name**	**N**	**Beta**	**95% CI**[1]	**p-value**
P04899	GNAI2	105	-2.6	-8.3, 3.0	0.4
Q9BRK3	MXRA8	109	-0.48	-4.1, 3.1	0.8
P40925	MDH1	103	0.93	-5.0, 6.9	0.8
O00391	QSOX1	118	2.5	-1.9, 7.0	0.3
P13796	LCP1	102	0.25	-1.8, 2.3	0.8
Q8IV08	PLD3	103	-3.7	-11, 3.5	0.3
P09211	GSTP1	100	8.3	2.5, 14	**0.005**
P32119	PRDX2	102	-2.9	-9.1, 3.4	0.4
P30530	AXL	105	1.6	-3.1, 6.3	0.5
P15311	EZR	117	-4.0	-9.7, 1.8	0.2
P58499	FAM3B	97	-1.0	-6.8, 4.7	0.7
P14384	CPM	104	2.5	-3.3, 8.3	0.4
P11142	HSPA8	118	3.1	-2.5, 8.8	0.3
P00352	ALDH1A1	117	-2.1	-6.6, 2.3	0.3
P02751	FN1	118	8.0	1.9, 14	**0.011**
P51654	GPC3	113	0.87	-3.2, 4.9	0.7
P00966	ASS1	106	0.60	-2.6, 3.8	0.7
P26992	CNTFR	100	-3.2	-8.6, 2.3	0.3
Q6UX73	C16orf89	115	3.8	-0.95, 8.5	0.12
P09668	CTSH	107	5.2	0.26, 10	**0.039**
P17174	GOT1	116	-2.8	-8.6, 2.9	0.3
Q12860	CNTN1	117	0.60	-3.3, 4.5	0.8
O75351	VPS4B	104	3.7	-1.7, 9.0	0.2
P42785	PRCP	103	-2.9	-9.8, 4.1	0.4
Q8WZ75	ROBO4	114	0.58	-3.0, 4.1	0.7
Q9H0W9	C11orf54	113	-2.8	-7.4, 1.9	0.2
P63000	RAC1	97	1.4	-5.6, 8.3	0.7
Q12794	HYAL1	113	0.76	-4.1, 5.7	0.8
P37837	TALDO1	99	-1.4	-4.1, 1.3	0.3
P00751	CFB	101	2.4	-0.50, 5.2	0.10
P06727	APOA4	105	1.2	-1.3, 3.7	0.4

**Protein ID**	**Gene Name**	**N**	**Beta**	**95% CI**[1]	**p-value**
Q9BYE9	CDHR2	116	-2.9	-7.0, 1.2	0.2
Q5JS37	NHLRC3	94	-4.1	-9.2, 1.0	0.11
P04406	GAPDH	108	1.7	-4.1, 7.5	0.6
O14773	TPP1	116	0.95	-4.0, 5.9	0.7
P84077	ARF1	100	10	2.6, 18	**0.009**
Q03154	ACY1	99	0.98	-2.5, 4.4	0.6
P60953	CDC42	110	5.9	1.2, 11	**0.014**
Q13621	SLC12A1	115	-0.69	-5.2, 3.9	0.8
O43505	B4GAT1	107	-0.35	-5.2, 4.5	0.9
Q99519	NEU1	99	-5.2	-11, 0.76	0.087
O43490	PROM1	115	-1.4	-6.2, 3.4	0.6
Q9HB40	SCPEP1	102	-1.7	-6.6, 3.3	0.5
Q9H444	CHMP4B	100	1.0	-4.7, 6.8	0.7
O43451	MGAM	116	0.67	-3.0, 4.3	0.7
O00462	MANBA	117	-0.43	-3.9, 3.1	0.8
Q8NBJ4	GOLM1	106	-1.9	-4.8, 0.98	0.2
P19801	AOC1	107	0.53	-2.8, 3.8	0.8
P0DMV9	HSPA1B	118	2.7	-3.0, 8.4	0.3
O43895	XPNPEP2	103	-0.10	-3.7, 3.5	>0.9
Q9HBB8	CDHR5	113	-1.6	-6.3, 3.2	0.5
P00491	PNP	112	-2.1	-7.2, 3.1	0.4
Q13228	SELENBP1	115	-0.29	-5.6, 5.0	>0.9
O75874	IDH1	113	-1.8	-6.2, 2.6	0.4
P16152	CBR1	112	1.3	-3.8, 6.4	0.6
Q96KP4	CNDP2	112	0.16	-3.4, 3.7	>0.9
P08195	SLC3A2	115	-0.52	-7.4, 6.3	0.9
Q16651	PRSS8	95	-2.2	-9.3, 4.8	0.5
P51688	SGSH	101	0.01	-5.3, 5.3	>0.9
Q15907	RAB11B	106	-0.29	-7.6, 7.0	>0.9
P55287	CDH11	97	-1.8	-4.5, 0.95	0.2
Q14914	PTGR1	113	-0.57	-6.2, 5.1	0.8

Protein ID	Gene Name	N	Beta	95% CI[1]	p-value
P14550	AKR1A1	109	1.9	-2.1, 5.9	0.3
P51149	RAB7A	101	2.8	-5.2, 11	0.5
Q9H6X2	ANTXR1	102	-1.2	-5.6, 3.2	0.6
P43121	MCAM	111	-3.9	-8.3, 0.39	0.074
P52209	PGD	109	1.4	-2.5, 5.2	0.5
Q8NCC3	PLA2G15	106	-0.21	-5.7, 5.3	>0.9
P40121	CAPG	112	1.8	-3.9, 7.6	0.5
Q9HCN6	GP6	96	-1.8	-7.1, 3.5	0.5
P12277	CKB	103	3.6	-1.2, 8.4	0.14
P34059	GALNS	105	-0.87	-5.8, 4.0	0.7
Q96PD5	PGLYRP2	107	-1.4	-4.7, 1.9	0.4
P68371	TUBB4B	112	3.6	-0.90, 8.1	0.12
P29992	GNA11	102	2.1	-3.3, 7.6	0.4
P11021	HSPA5	115	1.7	-3.3, 6.6	0.5
P14543	NID1	95	-0.93	-5.2, 3.4	0.7
P06744	GPI	115	3.0	-1.6, 7.6	0.2
P51148	RAB5C	100	1.7	-5.6, 9.0	0.6
P34896	SHMT1	104	0.13	-4.0, 4.3	>0.9
Q3LXA3	DAK	109	0.28	-3.4, 4.0	0.9
Q9UGT4	SUSD2	103	2.4	-3.9, 8.6	0.5
O00241	SIRPB1	101	-0.87	-6.0, 4.3	0.7
P54760	EPHB4	111	-3.3	-9.0, 2.3	0.2
P08697	SERPINF2	112	-1.9	-6.0, 2.2	0.4
P61106	RAB14	111	-0.40	-8.8, 8.0	>0.9
Q8NHP8	PLBD2	108	1.3	-5.7, 8.4	0.7
P55017	SLC12A3	112	2.9	-1.5, 7.3	0.2
P55290	CDH13	113	0.43	-5.3, 6.1	0.9
P19827	ITIH1	112	2.6	-0.86, 6.2	0.14
P09603	CSF1	107	-3.3	-9.7, 3.1	0.3
Q92520	FAM3C	104	1.4	-4.5, 7.3	0.6
P50395	GDI2	109	3.5	-1.9, 8.9	0.2

Protein ID	Gene Name	N	Beta	95% CI[1]	p-value
Q08345	DDR1	109	-7.0	-14, 0.23	0.058
Q14393	GAS6	117	-0.42	-6.0, 5.1	0.9
O00533	CHL1	110	-0.92	-4.5, 2.7	0.6
P48594	SERPINB4	104	-1.1	-3.7, 1.5	0.4
Q9Y2E5	MAN2B2	113	0.05	-3.7, 3.8	>0.9
Q08174	PCDH1	95	-0.29	-4.1, 3.5	0.9
P04180	LCAT	109	-4.8	-10, 0.44	0.072
P07900	HSP90AA1	111	2.2	-1.3, 5.7	0.2
P06280	GLA	110	-6.4	-12, -0.34	**0.039**
P05546	SERPIND1	95	3.3	-0.05, 6.6	0.053
Q99816	TSG101	101	3.4	-1.5, 8.3	0.2
Q8IUL8	CILP2	117	-1.9	-8.3, 4.5	0.6
O75487	GPC4	110	2.3	-2.7, 7.3	0.4
P18085	ARF4	98	2.7	-3.4, 8.8	0.4
P08582	MFI2	111	-0.70	-5.6, 4.2	0.8
Q9UKU6	TRHDE	112	2.9	-2.2, 8.0	0.3
P20711	DDC	102	1.8	-1.9, 5.6	0.3
Q12913	PTPRJ	114	-2.1	-6.5, 2.3	0.3
Q9UIB8	CD84	109	-0.75	-5.7, 4.2	0.8
P35052	GPC1	109	0.96	-6.0, 7.9	0.8
Q8N271	PROM2	109	-2.4	-8.1, 3.3	0.4
Q9H6S3	EPS8L2	101	-0.86	-4.8, 3.1	0.7
Q96NY8	PVRL4	96	-4.8	-11, 1.2	0.12
Q6UVK1	CSPG4	113	0.22	-4.4, 4.8	>0.9
P02748	C9	95	1.7	-2.6, 6.0	0.4
P20073	ANXA7	110	3.6	-1.9, 9.1	0.2
P31946	YWHAB	96	-2.8	-9.4, 3.7	0.4
P50502	ST13	104	-1.6	-7.6, 4.4	0.6
O95497	VNN1	98	-3.4	-8.8, 1.9	0.2
O00299	CLIC1	99	0.44	-7.2, 8.1	>0.9
Q9HAT2	SIAE	102	-8.4	-16, -1.2	**0.023**

**Protein ID**	**Gene Name**	**N**	**Beta**	**95% CI[1]**	**p-value**
P08183	ABCB1	114	0.09	-4.4, 4.6	>0.9
Q12929	EPS8	106	-0.56	-4.5, 3.3	0.8
Q9H223	EHD4	106	0.93	-3.4, 5.3	0.7
P50895	BCAM	110	-1.8	-7.3, 3.8	0.5
P07384	CAPN1	98	-2.1	-5.3, 1.1	0.2
P05023	ATP1A1	115	5.0	0.35, 9.6	**0.035**
O43493	TGOLN2	107	-4.6	-8.3, -0.83	**0.017**
Q00796	SORD	94	2.0	-3.6, 7.7	0.5
P15313	ATP6V1B1	95	0.02	-4.0, 4.0	>0.9
O43707	ACTN4	109	1.4	-1.6, 4.5	0.3
P38606	ATP6V1A	101	3.0	-1.2, 7.1	0.2
Q9Y6W3	CAPN7	108	-0.15	-3.8, 3.5	>0.9
Q5JWF2	GNAS	100	-3.6	-12, 4.6	0.4
P12821	ACE	109	-1.1	-5.7, 3.5	0.6
Q86YQ8	CPNE8	102	1.9	-5.3, 9.1	0.6
P08572	COL4A2	109	2.9	-2.0, 7.8	0.2
P29401	TKT	98	3.8	0.25, 7.3	**0.036**
O00159	MYO1C	106	-0.97	-5.0, 3.1	0.6
P61019	RAB2A	98	0.01	-7.9, 8.0	>0.9
Q16706	MAN2A1	109	2.5	-2.0, 7.0	0.3
P35241	RDX	103	-0.82	-5.8, 4.1	0.7
Q9BYF1	ACE2	108	0.48	-5.9, 6.8	0.9
Q92673	SORL1	115	-2.9	-7.9, 2.1	0.2
Q13332	PTPRS	115	1.9	-2.1, 5.8	0.4
P55786	NPEPPS	99	0.17	-5.0, 5.3	>0.9
P54753	EPHB3	109	-2.4	-9.2, 4.5	0.5
Q7Z7M0	MEGF8	118	0.24	-5.4, 5.9	>0.9
P36871	PGM1	98	2.7	-1.6, 7.0	0.2
Q92896	GLG1	101	1.4	-3.4, 6.3	0.6
P13797	PLS3	104	-0.23	-5.9, 5.5	>0.9
P0C0L4	C4A	110	2.2	-1.0, 5.5	0.2

Protein ID	Gene Name	N	Beta	95% CI[1]	p-value
P12111	COL6A3	116	-0.68	-4.0, 2.6	0.7
O75339	CILP	106	-2.3	-7.5, 2.9	0.4
Q9UQB8	BAIAP2	98	-1.5	-5.8, 2.7	0.5
P18206	VCL	105	-2.4	-6.1, 1.2	0.2
Q5SZK8	FREM2	108	1.1	-2.2, 4.5	0.5
O00468	AGRN	106	0.55	-3.7, 4.8	0.8
Q99523	SORT1	101	-8.5	-15, -1.6	**0.017**
P23470	PTPRG	98	-3.2	-8.8, 2.3	0.3
P22314	UBA1	98	4.5	0.35, 8.6	**0.034**
Q14126	DSG2	96	-6.9	-13, -1.2	**0.019**
P32004	L1CAM	105	-3.5	-7.9, 0.84	0.11
Q6V0I7	FAT4	107	0.51	-2.5, 3.5	0.7
P22105	TNXB	104	0.60	-4.9, 6.1	0.8
Q09666	AHNAK	96	-2.2	-4.1, -0.38	**0.019**
P11717	IGF2R	99	-2.8	-8.0, 2.3	0.3

[1]CI = Confidence Interval
